# Severity of Disease and COVID-19 Complications During Hospital Stay: A Prospective Cohort Study

**DOI:** 10.34172/aim.2022.62

**Published:** 2022-06-01

**Authors:** Ramin Sami, Forogh Soltaninejad, Azin Shayganfar, Sam Mirfendereski, Marjan Mansourian, Nilufar Khademi, Mehrnegar Dehghan, Zahra Khorrami, Soheila Jalali, Zeinab Mokhtari

**Affiliations:** ^1^Department of Internal Medicine, School of Medicine, Isfahan University of Medical Sciences, Isfahan, Iran; ^2^Department of Radiology, School of Medicine, Isfahan University of Medical Sciences, Isfahan, Iran; ^3^Biostatistics, Pediatric Cardiovascular Research Center, Cardiovascular Research Institute, Isfahan University of Medical Sciences, Isfahan, Iran; ^4^School of Medicine, Isfahan University of Medical Sciences, Isfahan, Iran; ^5^Ophthalmic Epidemiology Research Center, Research Institute for Ophthalmology and Vision Science, Shahid Beheshti University of Medical Sciences, Tehran, Iran; ^6^Department of Epidemiology and Biostatistics, School of Health, Isfahan University of Medical Sciences, Isfahan, Iran; ^7^Food Security Research Center, Isfahan University of Medical Sciences, Isfahan, Iran

**Keywords:** COVID-19, ICU admission, Mortality, SARS-CoV-2, Severity

## Abstract

**Background:**

COVID-19, with its high transmission and mortality rates and unknown outcomes, has become a major concern in the world. Among people with COVID-19, severe cases can quickly progress to serious complications, and even death. So, the present study aimed to examine the relationship between the severity of the disease and the outcome in patients afflicted by COVID-19 during hospitalization.

**Methods:**

A total of 653 patients with COVID-19 aged 18 years or older were included from Khorshid hospital in Isfahan, Iran and followed for a mean of 22.72 days (median 23.50; range 1–47). Severe COVID-19 was defined by respiration rate≥30 times/min, oxygen saturation level≤88% in the resting position, and pulse rate≥130/min. The primary outcome was mortality. The secondary outcomes included need for mechanical ventilation and intensive care unit (ICU) admission.

**Results:**

During 4233 person-days of follow-up, 49 (7.5%) deaths, 27 (4.1%) invasive ventilation and 89 (13.6%) ICU admissions in hospital were reported. After adjustment for potential confounders, severity of the disease was positively associated with risk of mortality, invasive ventilation and ICU admissions (hazard ratio [HR]: 5.99; 95% CI: 2.85, 12.59; *P*<0.001, HR: 7.09; 95% CI: 3.24, 15.52; *P*<0.001 and HR: 4.88; 95% CI: 2.98, 7.98; *P*<0.001, respectively). In addition, greater age (HR=1.04; 95% CI=1.02-1.07; *P*=0.002), chronic kidney disease (HR=3.05; 95% CI=1.35, 6.90; *P*=0.008), blood urea nitrogen (BUN) (HR=1.04; 95% CI=1.03–1.05; *P*<0.001) and creatinine (HR=1.44; 95% CI=1.26-1.65; *P*<0.001) were probably significant risk factors for mortality in severe COVID-19 patients.

**Conclusion:**

More intensive therapy and special monitoring should be implemented for patients with older age, hypertension and kidney disease who are infected with COVID-19 to prevent rapid worsening.

## Introduction

 Severe acute respiratory syndrome coronavirus 2 (SARS-CoV-2), with its high transmission and mortality rates and unknown outcomes, has become a major concern in the world.^1^ About the end June 2022, according to the World Health Organization (WHO), more than 537 million people were infected with the virus worldwide, with nearly 6 319 395 deaths in the world.^2^ Although most people with COVID-19 have a mild form of the disease and a good prognosis, severe cases can quickly progress to pneumonia, acute distress syndrome, multiple organ dysfunction, and even death.^3,4^ Besides, complications such as heart disease,^5^ arterial thromboembolism,^6,7^ encephalopathy,^8^ and other neurological manifestations^9^ have been observed in these patients. Currently, no approved specific and effective treatment is available for COVID-19, and patients receive symptomatic and supportive treatment.^10^

 The outcome and presentations of this infectious disease depend on different factors. The findings of a systematic review revealed that underlying diseases, including hypertension, cardiovascular disease, and diabetes, as well as the presence of acute inflammation and abnormal kidney and liver function tests, are associated with increased risk of mortality with COVID-19.^11^ Additionally, male gender, higher age, and morbid obesity may contribute to a poorer clinical outcome.^12-14^ In addition to individual characteristics, the disease complications may be affected by the effects of COVID-19 on healthcare systems.^15,16^

 Due to the recent emergence of the novel coronavirus, our understanding of the complications and end-points of SARS-CoV-2, especially in the Middle East, is still limited. Moreover, the existing evidence has shortcomings due to the small sample size of the studies and the lack of follow-up of the patients. Furthermore, the complications of SARS-CoV-2 impose a huge burden on the healthcare system, especially in developing countries that face critical shortages in terms of healthcare staff and facilities. Therefore, understanding the consequences associated with this viral infection, especially in its severe form, would help in finding effective treatments for this disease, preventing its acute complications, reducing its mortality, and effectively prioritizing healthcare resources. Hence, the present study aimed to examine the relationship between the severity of the disease and the outcome in patients afflicted by COVID-19during hospitalization.

## Methods and Materials

###  Study Population

 This was a prospective, single-center cohort study. The Khorshid COVID Cohort (KCC) study was initiated in February 2020, at Khorshid hospital in Isfahan, Iran. Khorshid Hospital is the referral center for adults suffering from COVID-19 in Isfahan. The design of the KCC has been reported previously.^17^ The follow-up flow chart is presented in Supplementary file 1 (Figure S1). All adult patients (≥ 18 years old) who were diagnosed with COVID-19 based on positive results of reverse transcription polymerase chain reaction (RT-PCR) of pharyngeal swab samples or typical lesions of the disease on chest CT scans^18^ were recruited. The exclusion criteria were patients refusing to participate in the study, pregnant or breastfeeding women, and prison inmates. Eligible patients were divided into two groups according to the severity of the disease. Based on the consensus of clinicians and previous evidence,^19^ the diagnosis of severe disease would be determined if all the criteria including respiration rate (RR) ≥ 30 times/min, oxygen saturation level ≤ 88% in the resting position, and pulse rate ≥ 130/min were met.

###  Clinical and Paraclinical Assessment

 At the baseline, information on demographics, medical history, and underlying chronic diseases was obtained from the medical records of the patients using an organized questionnaire. Body temperature, RR, oxygen saturation, heart rate, systolic blood pressure (SBP), diastolic blood pressure (DBP), weight, and height of the patients were evaluated on admission to the emergency ward by trained nurses. Routine blood examinations including complete blood count with differential, hemoglobin (Hb), liver functional tests (alanine aminotransferase [ALT] and aspartate aminotransferase [AST]), lactate dehydrogenase (LDH), inflammatory factors (ferritin, C-reactive protein [CRP], erythrocyte sedimentation rate [ESR]), blood urea nitrogen (BUN), creatinine, and arterial blood gases (PH, HCO_3_, PCO_2_) were measured at the baseline for each person. A total of 436 patients underwent pulmonary CT scanning by a 64-slice Philips scanner according to the protocol of low dose and non-contrast chest CT. Images were analyzed by two experienced radiologists. Diagnosis of suspected patients was made based on the Radiological Society of North America (RSNA) guidelines.^20,21^ For quantifying the results of lung abnormalities on CT scan, a thin-section CT score was calculated based on the extent of involved areas.^22^

###  Follow-up and Improvement Ascertainment

 All participants were followed until discharge from the hospital or death. During a mean follow-up of 22.72 days, (median 23.50; range 1–47), all of the participants completed follow-up. The primary outcome was mortality after hospital admission. The secondary outcomes included need for mechanical ventilation and admission to the intensive care unit (ICU). Discharge criteria were defined as respiratory rate < 20, pulse rate < 100, oxygen saturation > 92% in the resting state, normal body temperature (< 38℃) for more than two days without any antipyretic treatments, and the ability to normally swallow solid oral medication. However, some patients stayed in the hospital up to 14 days after the onset of the symptoms because appropriate care conditions and suitable provisions for quarantine at home could not be ensured.^23^ Any reported death was verified by a physician’s visit. Hospitalization duration was determined in days (from the date of hospital admission to the date of discharge).

###  Statistical Analysis

 According to the severity of the disease, participants were divided into two groups of severe and non-severe disease. The participants’ characteristics were compared between the two groups using the independent *t *test for continuous variables and the chi-square test for categorical ones. Multivariable logistic regression analysis was used to assess the relationship between baseline characteristics and the severity of COVID-19. Furthermore, in order to test for the association between the severity of the disease and the risk of mortality, mechanical ventilation, and admission to ICU, Cox proportional hazards regression models were constructed to estimate hazard ratios (HRs) and 95% confidence intervals (CIs). In the multivariable model, the HRs were adjusted for potential confounders, including age in years (continuous), gender (male, female), education (illiterate, high-school graduate, college graduate), systolic blood pressure, history of hypertension (yes, no), CT score (continuous) and other continuous covariates (white blood cell count, neutrophil and lymphocyte counts, AST, ferritin, CRP, ESR, BUN, and creatinine). An *P* value less than 0.2 in the crude model for these variables was considered as a criterion for adjusting potential confounding factors in the adjusted model. Follow-up time was considered for each patient from the date they were recruited into the study until the date of death, failure to follow-up, discharge from the hospital, or the end of follow-up (April 30, 2020), whichever happened first. All statistical analyses were performed using Stata version 14 (Stata Corp LLC; College Station, TX, USA). The statistical significance level in all tests was *P* ≤ 0.05, with two-tailed statistical analysis.

## Results

 A total of 653 patients with COVID-19 were included in the study. Of these, 604 (92.5%) were discharged, 49 (7.5%) died in hospital, 27 (4.1%) needed invasive ventilation and 89 (13.6%) were admitted to the ICU. Almost a third of the patients (35.1%) were identified as severe cases and 64.9% were non-severe cases on admission. The incidence rates of mortality, required intubation and ICU admission were 1.68, 1.14 and 12.83 per 1000 person-days, respectively. The mean age (SD) of participants at the baseline was 57.26 (15.48) years. Overall, 61.1% were men. The baseline characteristics of the participants are shown in Table 1. Severe patients were older and more likely to have lower education and higher percent of CT score for severity in comparison to those with non-severe disease. Furthermore, hypertension was the most common co-morbidity (34.9%) in all patients and significantly more frequent in the severe cases compared with non-severe patients (100 (43.7%) vs 128 (30.2%), *P***<**0.001). The mean systolic blood pressure, white blood cell count, neutrophil and lymphocyte counts, liver tests (AST and LDH), inflammatory markers (ferritin, CRP and ESR), kidney tests (BUN and creatinine), and hospitalization days were significantly higher in severe patients with COVID-19 than non-severe cases. Furthermore, a significant decrease was observed in hemoglobin levels in severe subjects. Moreover, the prevalence rates of death, invasive ventilation, and ICU admission were significantly higher in severe cases compared to non-severe patients **(**Table 1).

**Table 1:** Characteristics of Hospitalized Patients with COVID-19 in the Khorshid Cohort Study^a^

**Characteristic**	**All Patients**	**Severe**	**Non-severe**	***P *** **Value ** ^b^
N (%)	653 (100)	229 (35.1)	424 (64.9)	
Gender				0.556
Female, n (%)	254 (38.9)	93 (36.6)	161 (63.4)	
Male, n (%)	399 (61.1)	136 (34.1)	263 (65.9)	
Age (year), Mean ± SD	57.26 ± 15.48	63.75 ± 14.34	53.77 ± 14.95	< 0.001
BMI (kg/m^2^), Mean ± SD	27.42 ± 3.26	27.23 ± 3.08	27.52 ± 3.35	0.274
Normal weight, n (%) ^c^	90 (13.8)	36(40.0)	54 (60.0)	0.342
Overweight and Obese n (%)	556 (85.1)	192(34.5)	364 (65.5)	
Smoker (Current), n (%)	72 (11.0)	22(30.5)	50 (69.5)	0.146
Education, n (%)				0.004
Illiterate	110 (16.8)	48 (43.6)	62 (56.4)	
High-school graduate	59 (9.0)	16 (27.1)	43 (72.9)	
College graduate	54 (8.3)	13 (24.1)	41 (75.9)	
Temperature on admission (categorized, > 38℃), n (%)	91 (13.9)	27 (29.7)	64 (70.3)	0.287
Systolic blood pressure (mm Hg), Mean ± SD	132.90 ± 19.63)	134.69 ± 20.48)	131.94 ± 19.12)	0.031
Diastolic blood pressure (mm Hg), Mean ± SD	81.86 ± 28.27)	82.39 ± 44.83)	81.58 ± 12.07)	0.717
CT score for severity, Mean ± SD	10.12 ± 5.02	12.33 ± 5.55	9.17 ± 4.45	< 0.001
Co-morbidities (Yes)				
History of diabetes, n (%)	181 (27.7)	69 (38.1)	112 (61.9)	0.315
History of hypertension, n (%)	228 (34.9)	100 (43.9)	128 (56.1)	< 0.001
Cardiovascular disease, n (%)	131 (20.1)	53 (40.5)	78 (59.5)	0.154
Respiratory disease, n (%)	71 (10.9)	31 (43.7)	40 (56.3)	0.115
Chronic kidney disease, n (%)	36 (5.5)	17 (47.2)	19 (52.8)	0.149
Biochemistry parameters				
White blood cell count (× 10^9^/L), Mean ± SD	6074.47 ± 3059.31	6895.13 ± 3818.51	5630.43 ± 2449.42	< 0.001
Neutrophil count (%), Mean ± SD	73.19 ± 11.49	78.07 ± 9.87	70.53 ± 11.44	< 0.001
Lymphocyte count (%), Mean ± SD	20.87 ± 9.89	16.73 ± 8.75	23.12 ± 9.75	< 0.001
Platelet count (× 10^9^/L), Mean ± SD	188.37 ± 71.52	188.91 ± 77.13	188.07 ± 68.38	0.888
Hemoglobin (g/dL), Mean ± SD	13.21 ± 1.85	12.93 ± 2.03	13.37 ± 1.72	0.006
Liver tests				
ALT (U/L), Mean ± SD	33.32 ± 45.57	32.87 ± 37.33	33.57 ± 49.71	0.857
AST (U/L), Mean ± SD	46.46 ± 47.33	53.95 ± 69.98	42.21 ± 26.31	0.017
LDH (U/L), Mean ± SD	695.85 ± 898.71	929.30 ± 711.87	539.40 ± 225.40	0.028
Inflammatory factors				
Ferritin (ng/mL), Mean ± SD	548.65 ± 274.52	607.14(297.77	494.67 ± 241.05	0.021
CRP (mg/L), Mean ± SD	30.56 ± 22.31	36.59 ± 23.02	26.81 ± 21.04	< 0.001
ESR (ml/h), Mean ± SD	47.54 ± 27.51	53.10 ± 29.09	42.94 ± 25.94	< 0.001
Kidney tests				
BUN (mg/dL), Mean ± SD	19.96 ± 14.04	23.33 ± 16.74	18.11 ± 11.93	< 0.001
Cr (mg/dL), Mean ± SD	1.23 ± 1.09	1.36 ± 1.25	1.16 ± 0.99	0.040
Arterial blood gas				
PH, Mean ± SD	7.36 ± 0.06	7.36 ± 0.06	7.36 ± 0.06	0.947
HCO_3_ (mm Hg), Mean ± SD	23.65 ± 3.67	23.59 ± 3.75	23.69 ± 3.62	0.758
PCO_2_ (mm Hg), Mean ± SD	44.37 ± 9.77	44.18 ± 10.26	44.47 ± 9.50	0.721
Length hospital day, Mean ± SD	6.48 ± 5.64	8.60 ± 6.83	5.34 ± 4.50	< 0.001
SARS-CoV-2 nucleic acid test				
Positive, n (%)	450 (68.9)	178 (39.6)	272 (60.4)	
Negative, n (%)	140 (21.4)	36 (25.7)	104 (74.3)	
Without test-Positive chest CT scans, n (%)	63 (9.7)	15 (23.8)	48 (76.2)	
Outcomes				
Death, n (%)	49 (7.5)	40 (81.6)	9 (18.4)	< 0.001
Invasive ventilation, n (%)	27 (4.1)	25 (92.6)	2 (7.4)	< 0.001
ICU admission, n (%)	89 (13.6)	65 (73.0)	24 (27.0)	< 0.001

^a^ Values are expressed as means ± SD for continuous variables and percentages for categorical variables. ^b^ Independent *t *test for quantitative variables and χ2 test for qualitative variables. ^c^ Normal weight: BMI lower than 24.9, Overweight and obese: BMI more than 25.

 As shown in Table 2, ground glass opacity (GGO) with or without consolidation appeared in nearly half of all patients. The incidence of GGO in the severe group tended to be higher than non-severe participants (50.4% vs 41.1%, *P*= 0.076). However, the other morphology of lesion patterns (consolidation and crazy paving pattern) were similar in severe and non-severe groups. Pleural effusion was found in only 7.3% of participants which was significantly different in severe patients in comparison to non-severe patients (12.0% vs 5.2%, *P*= 0.01). Moreover, distribution of the lesions was noticed in lower lobes at 49.9% and in upper lobes at 7.9%, and involvement of lower lobes was significantly more in non-severe than severe subjects (53.0% vs 42.7%, *P*= 0.023). Additionally, a multifocal pattern was seen in 91.1% of all patients, which was higher in non-severe than severe cases (91.6 % vs 89.7%, *P*= 0.001).

**Table 2:** Baseline Radiographic Findings of Patients with COVID-19

**CT Findings**	**All Patients**	**Severe (n=133)**	**Non-severe (n=309)**	***P *** **Value ** ^a^
GGO				0.076
Negative	248 (56.1%)	66 (49.6%)	182 (58.9%)	
Positive	194 (43.9%)	67 (50.4%)	127 (41.1%)	
GGO consolidation				0.209
Negative	240 (54.9%)	79 (59.8%)	161 (52.8%)	
Positive	197 (45.1%)	53 (40.2%)	144 (47.2%)	
Consolidation				0.201
Negative	408 (93.6%)	127 (96.2%)	281 (92.4%)	
Positive	28 (6.4%)	5 (3.8%)	23 (7.6%)	
CN				0.093
Negative	413 (95.6%)	128 (97.7%)	285 (94.7%)	
Positive	19 (2.9%)	3 (2.3%)	16 (5.3%)	
BronWT				0.315
Negative	402 (93.1%)	121 (92.4%)	281 (93.4%)	
Positive	30 (6.9%)	10 (7.6%)	20 (6.6%)	
Ret				0.361
Negative	271 (62.3%)	77 (58.3%)	194 (64.0%)	
Positive	164 (37.7%)	55 (41.7%)	109 (35.0%)	
LAP				0.085
Negative	432 (99.8%)	130 (99.2%)	302 (100.0%)	
Positive	1 (0.2%)	1 (0.8%)	0 (0.0%)	
Vas Enlarge				0.145
Negative	410 (94.5%)	127 (96.2%)	283 (93.7%)	
Positive	24 (5.5%)	5 (3.8%)	19 (6.3%)	
Pleural Effusion				0.010
Negative	407 (92.7%)	117 (88.0%)	290 (94.8%)	
Positive	32 (7.3%)	16 (12.0%)	16 (5.2%)	
Distribution				0.023
None	183 (42.2%)	66 (50.4%)	117 (38.7%)	
Upper	34 (7.9%)	9 (6.9%)	25 (8.3%)	
Lower	216 (49.9%)	56 (42.7%)	160 (53.0%)	
Transverse				0.112
None	107 (27.2%)	43 (32.2%)	64 (23.5%)	
Peripheral	272 (69.0%)	73 (59.8%)	199 (73.0%)	
Central	15 (3.8%)	6 (4.9%)	9 (3.3%)	
Region				0.138
Unilateral	34 (8.3%)	5 (4.1%)	29 (10.0%)	
Bilateral	377 (91.7%)	117 (95.9%)	260 (90.0%)	
Scattered				0.001
Focal	19 (4.5%)	1 (0.8%)	18 (6.0%)	
Multifocal	386 (91.0%)	113 (89.7%)	273 (91.6%)	
Diffuse	19 (4.5%)	12 (9.5%)	7 (2.3%)	

^a^ Based on χ2 test, Fisher exact test, or Mann-Whitney U test. GGO, ground glass opacity; CN, centric nodular lesions; BronWT, bronchial wall thickening; Ret, reticular pattern; LAP, lymphadenopathy; Vas Enlarge, vascular enlargement.

 After multivariable adjustment, the chance of severe disease was augmented with increasing age (OR:1.04; 95% CI: 1.01, 1.08; *P*= 0.021), CT score (OR = 1.13; 95% CI = 1.03,1.24; *P*= 0.011), and history of hypertension (OR = 2.90; 95% CI = 1.15,7.34; *P*= 0.024). In addition, higher serum ferritin concentrations were associated with severe disease (OR = 1.01; 95% CI = 1.00,1.02; *P*= 0.010) (Table 3).

**Table 3:** Association between Different Potential Risk Factors and Severity of COVID-19 on Admission

**Variable**	**Unadjusted Model**		**Adjusted Model ** ^b^	
	**OR (CI 95%)**	***P *** **Value** ^a^	**OR (CI 95%)**	***P *** **Value** ^a^
Gender (Men), *n* (%)	1.12 (0.80,1.55)	0.509		
Age (year)	1.05 (1.04,1.06)	< 0.001	1.04 (1.01,1.08)	0.021
Normal weight, n (%) ^c^	Ref.	0.315		
Overweight and Obese n (%)	0.79 (0.50,1.25)			
Smoker (Current), n (%)	0.97 (0.56,1.65)	0.899		
Education, n (%)				
Illiterate	Ref.		Ref.	
High-school graduate	0.42 (0.22, 0.81)	0.010	0.50 (0.15, 1.64)	0.250
College graduate	0.36 (0.18, 0.72)	0.004	0.68 (0.20, 2.23)	0.519
Temperature on admission (categorized, > 38℃), n (%)	0.75 (0.46, 1.22)	0.246		
Systolic blood pressure (mm Hg)	1.01 (0.99, 1.02)	0.089	0.97 (0.95, 0.99)	0.023
Diastolic blood pressure (mm Hg)	1.0 (0.99, 1.01)	0.730		
CT score for severity n (%)	1.14 (1.09, 1.195)	< 0.001	1.13 (1.03, 1.24)	0.011
Co-morbidities (yes)				
History of diabetes, n (%)	1.20 (0.84, 1.71)	0.312		
History of hypertension, n (%)	1.79 (1.28, 2.50)	0.001	2.90 (1.15, 7.34)	0.024
Cardiovascular disease, n (%)	1.33 (0.90, 1.97)	0.153	0.86 (0.29, 2.56)	0.780
Respiratory disease, n (%)	1.50 (0.91, 2.48)	0.110	0.71 (0.13, 3.93)	0.697
Chronic kidney disease, n (%)	1.71 (0.87, 3.36)	0.120	1.08 (0.12, 9.78)	0.948
Biochemistry parameters				
White blood cell count (× 10^9^/L)	1.00 (1.00, 1.00)	< 0.001	1.00 (1.00, 1.00)	0.090
Neutrophil count (%)	1.07 (1.05, 1.09)	< 0.001		
Lymphocyte count (%)	0.92 (0.91, 0.94)	< 0.001		
Platelet count (× 10^9 /L)	1.0 (0.99, 1.01)	0.888		
Hemoglobin (g/dL)	0.88 (0.80, 0.96)	0.004	0.97 (0.74, 1.27)	0.799
Liver tests				
ALT (U/L)	1.0 (0.99, 1.01)	0.857		
AST (U/L)	1.01 (1.00, 1.02)	0.007	1.00 (0.98, 1.02)	0.953
LDH (U/L)	1.01 (1.00, 1.04)	< 0.001	1.00 (0.99, 1.01)	0.573
Inflammatory factors				
Ferritin (ng/mL)	1.01 (1.00, 1.02)	0.024	1.01 (1.00, 1.02)	0.010
CRP (mg/L)	1.02 (1.01, 1.03)	< 0.001	1.00 (0.96, 4.04)	0.948
ESR (mL/h)	1.01 (1.00, 1.02)	< 0.001	1.01 (0.97, 1.04)	0.691
Kidney tests				
BUN (mg/dL)	1.03 (1.01, 1.04)	< 0.001	1.06 (0.92, 1.21)	0.427
Cr (mg/dL)	1.17 (1.01, 1.35)	0.033	0.17 (0.01, 2.32)	0.183
Arterial blood gas				
PH	0.91 (0.06, 14.29)	0.945		
HCO_3_ (mm Hg)	0.99 (0.95, 1.04)	0.758		
PCO_2_ (mm Hg)	0.99 (0.98, 1.01)	0.721		

^a^Based on Logistic regression model. ^b^ The adjusted model was adjusted for age, education, systolic blood pressure, CT score for severity, comorbidities, white blood cell count, hemoglobin, AST, LDH, inflammatory factors, BUN and creatinine.

**Figure 1:**
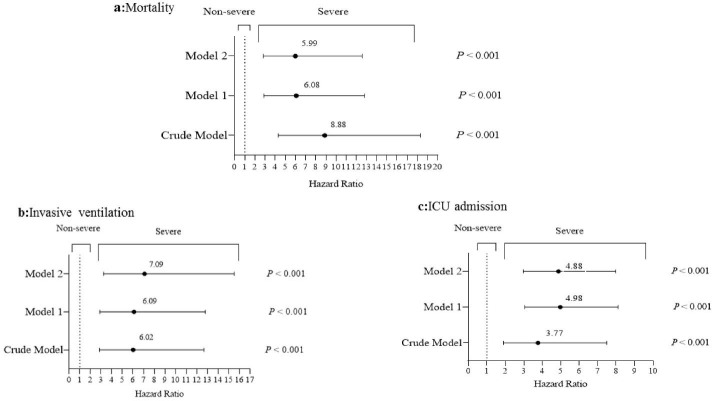
Hazard Ratios for Mortality, Ventilation Status and ICU Admissions According to Severity of Disease. Cox proportional hazards regression models for estimating HRs and 95% CIs. Model 1, multivariable adjusted for age and gender; Model 2, further controlled for, education, systolic blood pressure, history of hypertension, CT score, White blood cell count, neutrophil count, lymphocyte, AST, LDH, hemoglobin, ferritin, CRP, ESR, BUN and Cr. Data are presented as hazard ratio (95% CI).

 In the follow-up period, mortality rates for COVID-19 ranged from 17.5% (40/229) in severe cases to 2.1% (9/424) in non-severe patients; 10.9% (25) of severe cases and 0.5 % (2) of non-severe cases used the mechanical ventilation and 28.4% (65) in severe cases and 5.7% (24) in non-severe patients were admitted to the ICU. As shown in Figure 1, after controlling for potential confounders, severity of the disease was associated with increased risk of mortality (HR = 5.99; 95% CI = 2.85,12.59; *P* < 0.001), invasive ventilation (HR = 7.09; 95% CI = 3.24,15.52; *P* < 0.001) and ICU admission (HR = 4.88; 95% CI = 2.98,7.98; *P* < 0.001).

**Figure 2:**
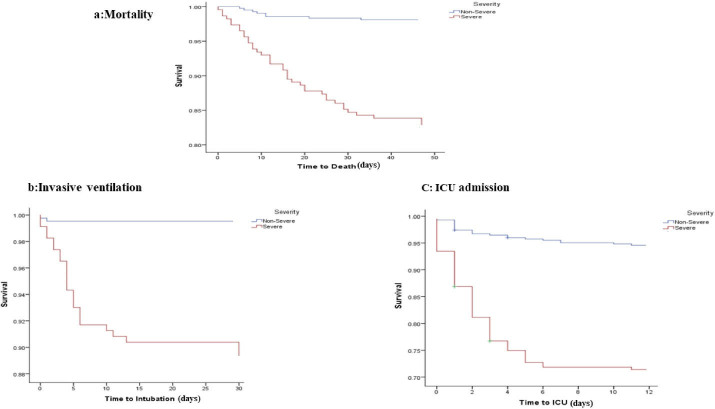
Kaplan-Meier Estimates of Survival Rate for Mortality, Ventilation Status and ICU Admissions According to Severity of Disease.

 As can be seen in Table 4, HRs for the association between severity of disease and risk of mortality indicated that age (HR = 1.04; 95% CI = 1.02–1.07;* P*= 0.002), chronic kidney disease (HR = 3.05; 95% CI = 1.35, 6.90;* P*= 0.008), BUN (HR = 1.04; 95% CI = 1.03–1.05;* P*< 0.001) and creatinine (HR = 1.44; 95% CI = 1.26–1.65;* P*< 0.001) were significant risk factors associated with death in cases with severe COVID-19. However, after adjusting for confounder variables, these disappeared. The Kaplan-Meier estimates of survival rate for all outcomes by severity/non-severity groups are shown in Figure 2.

**Table 4:** Hazard ratios^a^ for Mortality among Patients with Severe COVID-19

	**Unadjusted Model**		**Adjusted Model ** ^b^	
**Variable**	**HR (95% CI)**	***P *** **Value**	**HR (95% CI)**	***P *** **Value**
Gender (Men), n (%)	0.83(0.45, 1.55)	0.563		
Age (y)	1.04(1.02, 1.07)	0.001	0.99 (0.93,1.06)	0.750
Normal weight, n (%)	Ref.	0.290		
Overweight and Obese^c^, n (%)	1.75(0.62, 4.90)			
Smoker (Current), n (%)	0.93(0.21, 4.06)	0.923		
Temperature on admission (categorized, > 38℃), n (%)	1.38(0.58, 3.28)	0.470		
Systolic blood pressure (mm Hg)	1.00 (0.99, 1.02)	0.800		
Diastolic blood pressure (mm Hg)	0.98 (0.969, 1.00)	0.064	0.96 (0.91, 1.01)	0.135
Co-morbidities				
History of diabetes, n (%)	1.46 (0.77, 2.76)	0.251		
History of hypertension, n (%)	1.50 (0.81, 2.80)	0.197	1.93 (0.39, 9.54)	0.421
Cardiovascular disease, n (%)	0.34 (0.12, 0.97)	0.043	0.40 (0.06, 2.83)	0.360
Respiratory disease, n (%)	0.90 (0.35, 2.30)	0.827		
Chronic kidney disease, n (%)	3.05 (1.35, 6.90)	0.008	0.79 (0.09, 7.30)	0.836
Biochemistry Parameters				
White blood cell count (× 10^9/L)	1.00 (1.00, 1.00)	0.006	1.00 (1.00, 1.00)	0.077
Neutrophil count (%)	1.08 (1.03, 1.12)	0.001		
Lymphocyte count (%)	0.92 (0.87, 0.97)	0.002		
Platelet count (× 10^9 /L)	0.99 (0.99, 1.00)	0.020	0.99 (0.98, 1.01)	0.253
Hemoglobin (g/dL)	0.88 (0.76, 1.03)	0.106	0.89 (0.61, 1.32)	0.573
Liver tests				
ALT (U/L)	1.00 (0.99, 1.01)	0.870		
AST (U/L)	1.00 (1.00, 1.00)	0.551		
LDH (U/L)	1.00 (1.00, 1.00)	0.607		
Inflammatory factors				
Ferritin (ng/mL)	1.00 (1.00, 1.01)	0.030	1.00 (1.00, 1.02)	0.239
CRP (mg/L)	1.00 (0.98, 1.02)	0.962		
ESR (mL/h)	1.01 (1.00, 1.02)	0.295		
Kidney tests				
BUN (mg/dL)	1.04 (1.03, 1.05)	< 0.001	1.03 (0.96, 1.11)	0.405
Cr (mg/dL)	1.44 (1.26, 1.65)	< 0.001	0.60 (0.19, 1.87)	0.378

^a^ Cox proportional hazards regression models for estimating HRs and 95% CIs. ^b^ Adjusted for age, Diastolic blood pressure, Cardiovascular disease, Chronic kidney disease, White blood cell count, Platelet count, Hemoglobin, Ferritin, BUN and Cr. ^c^ Normal weight: BMI lower than 24.9, Overweight and obese: BMI more than 25.

## Discussion

 The results of this prospective cohort study showed 17.5% in-hospital mortality in patients with severe disease and 2.1% in those with non-severe disease during the mean 22.72 days of follow-up. We found that the severity of the disease was associated with a six-fold increase in the hazard of mortality and about a five- to seven-fold rise in the hazard of intubation and ICU admissionin in-hospital patients with COVID-19.Furthermore, higher age and comorbidities, especially diabetes, hypertension, and chronic kidney disease, were associated with increased odds of severity of the disease on admission and a higher risk of mortality in patients.

 To our knowledge, this is the first cohort study assessing the relationship between the severity of COVID-19 and mortality in the Middle East, with its unique composition of developing countries. Our findings are consistent with previous investigations, in which the severity of the disease was associated with increased risk of mortality and disease complications.^12^ The mortality rate was 17% in severe patients in our study, which differs from the results of previous studies.^12,24,25^ This inconsistency might be due to dissimilarities in baseline risks and diverse populations.

 Our findings are consistent with previous investigations^26^ in which about one-third of inpatients with COVID-19 suffered from hypertension and diabetes. Moreover, consistent with the results of the study by Li et al,^12^ after adjusting for potential confounders, hypertension was identified as a risk factor with a significant effect size correlating with disease severity in the present study. However, considering the inconclusiveness of the finding resulting from the wide confidence interval, more studies are warranted. Additionally, because the odds ratio was used as the effect size in this analysis, the strength of association might have been overestimated.^27^ These comorbidities were found to be associated with greater risk of disease severity of COVID-19; however, further studies are required to understand the mechanisms of this association.

 The biology of SARS-CoV-2 may have an important role in the increased risk of disease in some special comorbidities. Viral entry into the cell is accomplished by attaching to the cell via the angiotensin-converting-enzyme-2 (ACE2) receptor.^12,28^ The increased expression of ACE2 in patients with diabetes and hypertension could increase cellular binding affinity for the virus and facilitate its entry. Furthermore, reduced T cell function and dysregulation of pro-inflammatory cytokines, which is observed in diabetes and hypertension, might have a role in the severe manifestation of COVID-19.^29^

 Based on previous evidence, older age, hypertension, diabetes, and inflammatory proteins including ferritin could be considered risk factors associated with inpatient death.^11,14^ However, in the present study, unlike previous studies,^24^ only the comorbidity of hypertension had a considerable effect size for death after adjusting for confounding variables. Nonetheless, the results were inconclusive due to the wide confidence interval, which may have been caused by the small size of the severe group. In addition, no significant effect size was observed for other determinant factors in relation to mortality. It should be noted that the effect sizes reported in previous studies mostly did not involve controlling for potentially confounding factors. Besides, in those studies, risk factors were examined only in individuals with severe disease.

 Our study, in agreement with previous evidence,^12,30,31^ showed that older and male subjects seem to be more vulnerable to SARS-CoV-2; although these results are different from some previous investigations,^12,32^ age and sex indicated no substantial relationship with the risk of disease severity and mortality on admission. This discrepancy may be due to dissimilarities in the definition of disease severity. Because the severity variable is a composite measure, various definitions have been used for it in different studies. Also, the variable of age was categorical in those studies, while it is continuous in the present study.

 As previously reported,^33^ overweight and obesity are more prevalent in patients with COVID-19. The exact mechanism of the relationship between obesity and COVID-19 is unclear; however, inflammation induced by the adipose tissue and excessive levels of ACE2 in the adipose tissue as a potential reservoir for COVID-19 infection^33^ might contribute to this finding. Although in previous studies^13,24,34^ obesity, especially BMI more than 35, has been associated with critical endpoints of COVID-19 and death, in the present study, obesity was not linked to the severity and mortality of the disease. This inconsistency might be related to the fact that the number of people with BMI above 35 kg/m^2^ was small in our study.

 In the present study, consistent with previous studies,^35,36^ a considerable difference was observed between patients with severe and non-severe disease in terms of laboratory data, including hematologic, liver, and kidney tests and inflammatory factors. In concert with recent studies,^37,38^ among patients with severe illness, leukocytosis was more prevalent than leukopenia. Leukocytosis might be present in excessive inflammation, which also results in much higher ferritin, CRP, and ESR levels among severe cases. Furthermore, increased LDH levels that have been associated with poor prognosis, tissue injury, and infection,^39^ were observed in patients with severe disease. This finding might indicate lung injury and tissue damage in these patients. However, no considerable association was observed between biochemical markers other than creatinine concentration and the mortality risk. The findings of this study suggest that evaluation of laboratory markers on admission should be considered important in disease prognosis because of the differences observed in these markers in severe cases and the strong association between disease severity and critical outcomes and death.

 The lesion morphologies frequently associated with COVID-19 (GGO, consolidation, and crazy paving patterns) were also found in our study. In contrast to some studies which considered consolidation and crazy paving patterns to be more common in severe cases and GGO patterns to be more common in non-severe cases,^20,40,41^ the morphologic patterns of the lesions were the same in the two groups of patients in our study. Similar to the studies by Li et al^22^ and Yang et al,^42^ pleural effusion was seen much more frequently in subjects with severe disease than those with non-severe disease. It has been suggested that the occurrence of pleural effusion may be associated with poor prognosis in SARS-CoV-2.^43^ Furthermore, the distribution of the lesions was mainly detected in the lower lobes of the lungs compared to upper lobes, especially in non-severe patients. However, other studies have not mentioned this type of difference between the two groups and noted greater involvement of the lower lobes regardless of disease severity.^22,43^ This discrepancy may be due to the important time of imaging. According to previous evidence,^44,45^ GGO and other abnormality patterns usually develop between days 0 and 4 after symptom onset and peak at days 9–13. In the present study, a chest CT scan was performed on the admission day. Additionally, the findings of the present study showed a significant statistical difference between the CT scores of the patients with severe and non-severe disease. Our findings indicate that a higher CT score on admission can predict the severity of the disease and subsequent complications. However, as discussed earlier, the morphologic pattern of the lesions failed to predict disease severity contrary to some other studies. In summary, the CT scan can be very useful in both the diagnosis and prediction of the outcome in patients with COVID-19.

 The strengths of this study include its prospective nature, large sample size, and high participation rate. The limitations of the study include the fact that the use of medications for virus infection, which can affect disease outcome, was not adjusted for in the analysis. Furthermore, our assumption to consider discharged patients alive was another limitation. Moreover, given the observational nature of the study, the associations discovered do not necessarily constitute causality. Additionally, although potential confounders were adjusted for, residual and undetermined confounders might have had an impact on our results.

 In conclusion, the severity of the disease, defined based on clinical manifestations, was considerably associated with increased mortality, invasive ventilation, and ICU admission during hospital stay in Iran. This finding suggests that more intensive therapy and special monitoring should be implemented for patients with diabetes, hypertension, and kidney disease who are infected with COVID-19 to prevent rapid worsening.

## Supplementary Materials

Supplementary file 1 contains Figure S1.
