# Supplementary figures and images for: Severity of Disease and COVID-19 Complications During Hospital Stay: A Prospective Cohort Study
Source: Arch Iran Med. 2022 Jun 1;25(6):383–93. doi: 10.34172/aim.2022.62 (PMC11904273; doi:10.34172/aim.2022.62)

## Supplementary file 1

**Figure S1.** Cohort Study Flow Chart

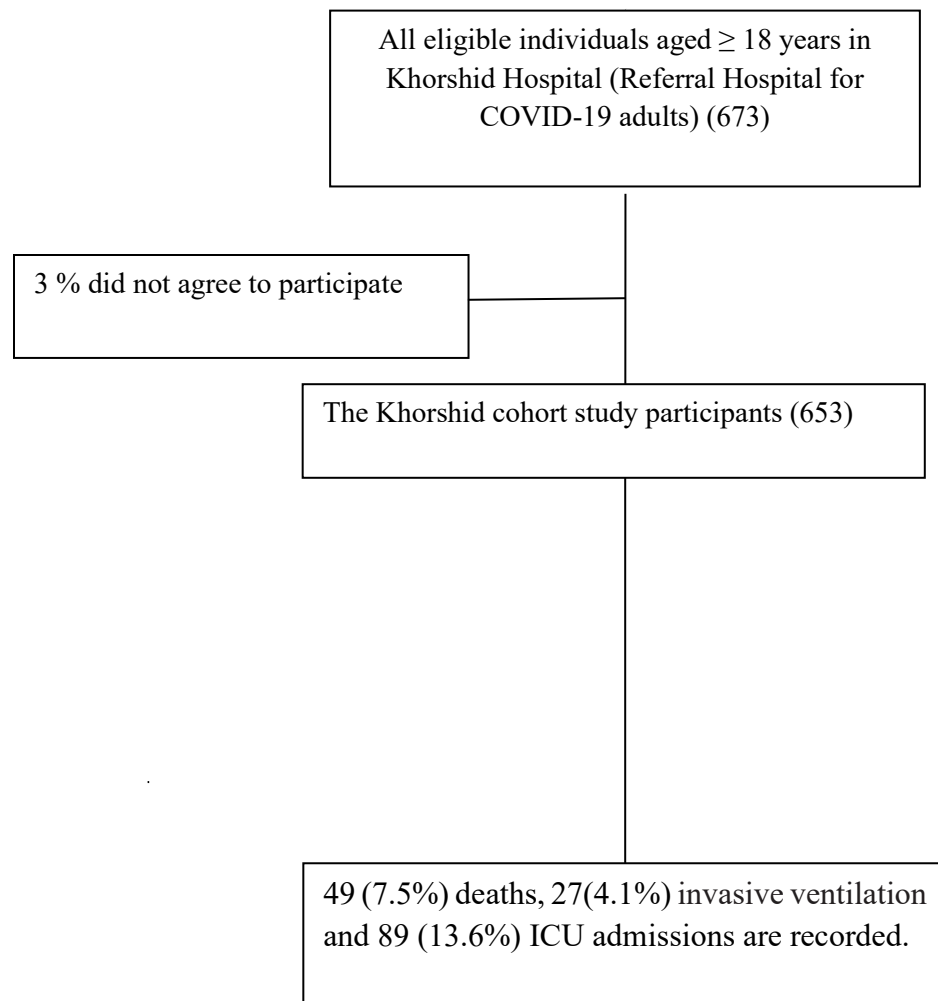

Supplement: Supplementary file 1 — contains Figure S1. [file aim-25-383-s001.pdf]
